# Neural dynamics of attention in HIV: A cognitive aging phenotype?

**DOI:** 10.1016/j.ebiom.2020.103114

**Published:** 2020-11-10

**Authors:** Sandeepa Sur, Leah H. Rubin

**Affiliations:** a Department of Radiology, Johns Hopkins University School of Medicine, Baltimore, MD USA; b Department of Neurology, Johns Hopkins University School of Medicine, Baltimore, MD USA; c Department of Psychiatry and Behavioral Sciences, Johns Hopkins University School of Medicine, Baltimore, MD USA; d Department of Epidemiology, Bloomberg School of Public Health, Johns Hopkins University, Baltimore, MD USA

Extensive efforts to minimize central nervous system (CNS) complications in virally suppressed people with HIV (PWH) have had limited success. The clinical presentation of CNS comorbidities such as HIV-associated neurocognitive disorders (HAND) is non-uniform and changes with age. This variability can impede the identification of the pathophysiological mechanisms underlying HAND. Focusing on specific cognitive systems, such as the attentional systems, may facilitate our understanding of the mechanisms leading to dysfunction and provide more narrowly defined treatment targets. In this issue of *EBioMedicine*, Arif and colleagues [1] aimed to identify the neural factors contributing to attention deficits in 79 PWH (28 with HAND) compared to 94 HIV-uninfected individuals. The clinical significance of a focus on attention is high, as deficits in this cognitive domain are central to cognitive aging in PWH [2] and can affect the ability to perform daily tasks such as poor HIV medication adherence [3]. The team employed a novel approach in the use of state-of-the art magnetoencephalography, (MEG) a functional neuroimaging method that measures magnetic field produced by electrical activity in the brain, to measure temporal and spatial cortical dynamics as measured by brain oscillations. The high temporal resolution of MEG is well-suited for attention tasks, which are a result of dynamic cortical interactions. Spatially, the resolution is almost as good as functional magnetic resonance imaging (fMRI; in millimeters) for cortical surfaces but is less adequate than fMRI for assessing “deep brain” or subcortical structures. The results provide compelling evidence that alterations in MEG-recorded oscillations in a number of brain regions reflect significant alterations in key aspects of attention and speed in PWH and the degree of alterations depends on age and HAND status.

A critical question that remains unanswered is whether HIV infection accelerates aging through mechanisms similar to aging process (accelerated aging), or HIV increases the risk for comorbidities, thereby accentuating the prevalence of disease at every age (accentuated aging) [4]. A variety of neuroimaging techniques have been used to identify biomarkers for accelerated aging by quantifying the interplay between age, comorbidities, HIV infection, and cognition. The findings are equivocal, with positive evidence for accelerated aging provided by structural magnetic resonance imaging (MRI) studies showing brain atrophy [5]. On the other hand, other neuroimaging studies show an independent effect of HIV and aging on brain volume (via 3-Dimensional Magnetization-Prepared Rapid Gradient-Echo Imaging [MPRAGE] sequence) [6], cerebral blood flow and brain activation (via fMRI) [7]. These conflicting findings could be ascribed to the fact that each imaging technique taps into a different neuropathologic or neurophysiologic aspect of brain change. Therefore, to clarify these findings, recent studies have investigated neural correlates of cognitive dysfunction relevant to PWH, with help of techniques such as MEG [8], with some focus on documenting attention- deficits (alerting and executive attention [9]). However, the brain mechanisms underlying attention-deficits in PWH remain poorly defined. Arif *et al.* [1] found that age differentially modulates the oscillatory dynamics underlying the attentional orienting processes in PWH and controls. They also reported an age-related increase in gamma oscillations in HAND vs. unimpaired PWH within three primary Ventral Attention Network nodes and attributed these findings to age-related compensation. However, these findings present only half the picture because the PWH groups under study report no comorbidities and are similar in HIV severity indices such as HIV-duration, years on antiretroviral medication, CD4 counts. Although unrepresentative of many PWH in whom comorbidities are common, these findings implicate age in the context of minimal comorbidities, as the main modulator for attention-deficits. Additionally, these findings seem to justify both accentuated aging (neural correlates of visual attention reorientation are susceptible to the effects of HIV disease) and accelerated aging models (the PWH level and pattern of functioning: compensation is observed in much older healthy adults).

Despite the obvious advantages of using the MEG technique, and having one of the largest MEG-datasets in PWH, there are several study limitations. First, aging was treated as a unidimensional concept, with limited understanding of how different physical and mental factors influence the course of PWH [10]. To identify at-risk individuals, aging needs to be treated as a multi-dimensional concept with various phenotypes; and phenotype-specific biomarkers could guide and assess treatments. Second, the generalizability of the findings is limited since the present study is comprised of primarily white, educated individuals with minimal comorbidities (i.e., substance use disorders). HIV disproportionately affects racial and ethnic minorities and gay and bisexual men, with the highest HIV transmission rate for blacks being 45.4%, followed by Latinos as 22.4%. Third, the study groups were significantly different in education and depression, both of which are well-known factors to influence cognitive function. Therefore, despite having controlled for education and depression status, caution should be taken while interpreting the ageing trajectories, which may not be immune to the effects of cognitive reserve and mental health.

Finally, like most studies examining comorbid and/or cognitive aging in PWH, this study design was cross-sectional. As a result, the temporal relationship between aging-related phenotypes is obscured. Longitudinal studies are needed to unobscure the actual cognitive trajectories, and in the process account for how specific comorbidities might contribute to other aging-related phenotypes. MEG measures are sensitive to brain dysfunction in PWH and can complement other approaches to provide useful information about PWH neuropathology, and aid in improving assessment, understanding and treatment of cognitive impairment in HIV as long as the samples are well characterized.

## Contributors

Sandeepa Sur: Literature search, commentary design, writing.

Leah Rubin: Commentary design, writing, editing.

## Declaration of Competing Interests

The authors declare no conflicts of interest.
